# The Roles of Biomarkers of Oxidative Stress and Antioxidant in Alzheimer's Disease: A Systematic Review

**DOI:** 10.1155/2014/182303

**Published:** 2014-05-14

**Authors:** Ya-Ting Chang, Wen-Neng Chang, Nai-Wen Tsai, Chih-Cheng Huang, Chia-Te Kung, Yu-Jih Su, Wei-Che Lin, Ben-Chung Cheng, Chih-Min Su, Yi-Fang Chiang, Cheng-Hsien Lu

**Affiliations:** ^1^Department of Neurology, Chang Gung Memorial Hospital-Kaohsiung Medical Center, Chang Gung University College of Medicine, 123 Ta Pei Road, Niao Sung District, Kaohsiung 833, Taiwan; ^2^Department of Biological Science, National Sun Yat-Sen University, Kaohsiung 80424, Taiwan; ^3^Department of Emergency Medicine, Chang Gung Memorial Hospital-Kaohsiung Medical Center, Chang Gung University College of Medicine, 123 Ta Pei Road, Niao Sung District, Kaohsiung 833, Taiwan; ^4^Department of Medicine, Chang Gung Memorial Hospital-Kaohsiung Medical Center, Chang Gung University College of Medicine, 123 Ta Pei Road, Niao Sung District, Kaohsiung 833, Taiwan; ^5^Department of Radiology, Chang Gung Memorial Hospital-Kaohsiung Medical Center, Chang Gung University College of Medicine, 123 Ta Pei Road, Niao Sung District, Kaohsiung 833, Taiwan

## Abstract

*Purpose*. Oxidative stress plays an important role in the pathogenesis of Alzheimer's disease (AD). This paper aims to examine whether biomarkers of oxidative stress and antioxidants could be useful biomarkers in AD, which might form the bases of future clinical studies. *Methods*. PubMed, SCOPUS, and Web of Science were systematically queried to obtain studies with available data regarding markers of oxidative stress and antioxidants from subjects with AD. *Results and Conclusion*. Although most studies show elevated serum markers of lipid peroxidation in AD, there is no sufficient evidence to justify the routine use of biomarkers as predictors of severity or outcome in AD.

## 1. Introduction

Alzheimer's disease is the most common form of dementia in the elderly and is characterized by a progressive loss of cognitive capacity and severe neurodegeneration. The pathophysiologic process is posited to be initiated by extracellular fibrillary *β*-amyloid deposition, with subsequent intraneuronal hyperphosphorylated tau protein aggregation [1]. Mutations in the amyloid precursor protein (APP), presenilin-1 (PS1), or PS2 that alter APP metabolism favor the production of a fibrillary form, A*β*. Such findings form the basis of the amyloid cascade hypothesis of AD pathophysiology [2].

**Figure 1:**
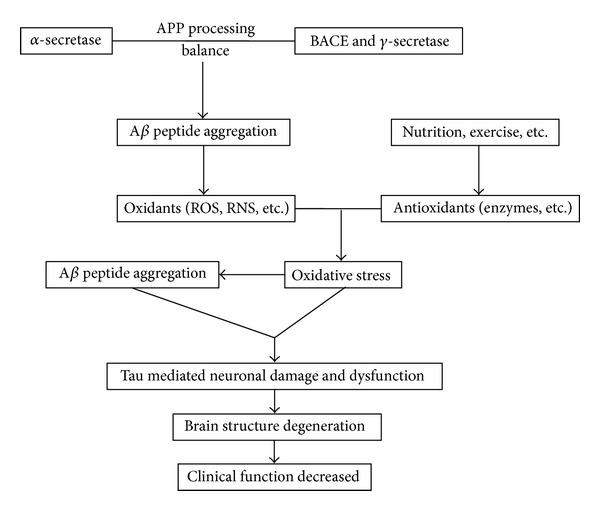
Oxidative stress in Alzheimer's dementia. APP: amyloid precursor protein; BACE: beta-secretase; ROS: reactive oxygen species; RNS: reactive nitrogen species; A*β*: amyloid *β*.

Although this amyloid cascade hypothesis may be the underlying pathogenesis for the familial form of AD, increasing evidence suggests that oxidative stress has a key role in late-onset sporadic forms, which are the majority of AD cases. Abnormal levels of oxidative stress have been reported in Alzheimer's disease in both the brain and blood stream [3, 4]. Changes in Alzheimer's disease that produce a prooxidative imbalance have been attributed to decrease in antioxidant defenses, toxicity related to amyloid-*β*, and/or altered metal metabolism in the brain and peripheral tissues [3, 4] (Figure 1).

Oxidative stress, a pathophysiologic imbalance between oxidants and antioxidants in favor of the former, with potential damage, has been shown in the blood, cerebrospinal fluid (CSF), and brain of neurologic patients with probable AD [5–12]. Biomarkers of oxidative stress in subjects with AD are classified as lipid peroxidation, protein oxidation, DNA oxidation, superoxide dismutase, and glutathione system [5, 13–16]. Biomarkers of oxidative damage to lipids include thiobarbituric acid-reactive substances (TBARS) and oxidized-LDL (ox-LDL) [7, 17]. The level of TBARS can be measured in plasma, serum, erythrocytes, and leukocytes [7], while ox-LDL is mostly measured in serum [17]. Oxidative attack on proteins results in the formation of protein carbonyls and protein nitration [18, 19]. Protein carbonyls and nitrated protein can be measured in plasma, serum, CSF, and brain tissue [18]. Regarding nucleic acids, 8-hydroxy-2-deoxyguanosine (8-OHdG) is one of the most commonly used markers of oxidative nucleic acid damage and can be measured in lymphocytes, leukocytes, and the brain [16].

The possible benefits of biomarkers in clinical practice include outcome prediction in AD patients that may further influence therapeutic regimens. The aim of this review is to determine whether biomarkers of oxidative stress can play an important prognostic role in the outcome of AD. The successful translation of these approaches to the clinics offers the promise of not only improving outcome prediction but also a more scientific basis for therapeutic options.

## 2. Methods

Studies were identified from a systematic search of PubMed, Scopus databases, Google Scholar, and the reference lists of all included studies and major relevant review papers. To find all of the relevant articles, PubMed was searched using the key words: “TBARS,” “oxidized LDL,” “protein carbonyls,” “8-HOG,” “antioxidant,” and “Alzheimer's disease” in various combinations. Case-control studies with human subjects were considered for inclusion. The articles selected were published in English between January 1985 and September 2013.

## 3. Results

### 3.1. Biomarkers of Lipid Peroxidation

Lipid peroxidation is one of the major consequences of oxidative imbalance-mediated injury to the brain. It causes changes in the fluidity and permeability of cell membranes and impairs the activity of membrane-bound enzymes. Lipid peroxidation also leads to the production of conjugated diene hydroperoxides and unstable substances that disintegrate into various aldehydes like malondialdehyde, 4-hydroxynonenal, and TBARS.

Several studies demonstrate that serum or plasma TBARS level in AD subjects is significantly higher than in controls [7, 13, 15, 20–28], while others observe no significant difference between AD subjects and controls [29–35] (Table 1). Results regarding erythrocyte TBARS level in AD are also controversial. Some studies observe higher erythrocyte TBARS levels in AD [5, 7, 31], while others observe no difference between AD and controls [6, 15, 29, 36]. A meta-analysis regarding blood TBARS level in AD and mild cognitive impairment reveals that TBARS levels are significantly elevated in Alzheimer's disease plasma/serum [37]. However, findings may vary by different patients selection criteria: some researchers observe AD subjects with MMSE 7–24 points do not have significantly higher serum TBARS than controls [34], while others observe AD subjects with 7–20 points as well as ADAS-cog 10–35 points have significantly higher serum TBARS than controls [20]. These suggest that MMSE alone is not enough to discriminate those with higher TBARS from those with lower TBARS. The lack of a link between MMSE and TBARS is also reported by another study, which suggests that MMSE is not correlated with TBARS [7], while plasma TBARS level may actually increase with the severity of cognitive dysfunction in AD [7].

Ox-LDL has been suggested to be produced by oxidized phospholipids released from brain tissue into circulation [38]. Ox-LDL is a promising marker of oxidative injury of the whole body. It may also be a peripheral marker that is linked to the severity of oxidative damage in the presence of dementia [23, 39, 40]. Serum ox-LDL level is universally higher in AD than in controls in the three studies.

### 3.2. Biomarkers of Protein Oxidation

Two different biomarkers of free-radical damage against protein have been suggested: protein oxidation that leads to the production of protein carbonyls [7, 14, 17, 18, 23, 28, 40–47] and protein peroxidation that leads to the production of nitrated protein [18, 19, 43, 46, 48–50] (Table 2). As a peripheral marker of oxidative stress in the brain, higher serum/plasma protein carbonyls level in AD is demonstrated in several studies despite varying patient selection criteria [7, 18, 40–43, 46]. Only two studies show no significant difference in serum/plasma protein carbonyls level between AD and controls [17, 23].

### 3.3. Biomarkers of Antioxidants

Antioxidants are suggested as potential indirect markers of oxidative stress processing in the brain of patients with AD (Table 3). Oxidative stress has been speculated to cause antioxidant consumption and thus, a decline in antioxidant levels [51]. Nonenzymatic compounds with antioxidant properties include vitamin A/carotenoids, vitamins C and E, and uric acid. On the other hand, antioxidant enzymes that vary on condition of oxidative stress in AD remain unsettled, since antioxidant enzymes like glutathione peroxidase (GPx) and superoxide dismutase (SOD) may be induced by oxidative stress (to increase their level or activity) or consumed (to decrease their level or activity) [37].

#### 3.3.1. Uric Acid

Three studies elaborate that plasma or serum uric acid level is significantly lower in AD [52–54], while three other studies do not observe this difference [29, 55, 56]. It is possible that excluding patients with metabolic syndrome plays an important role in measurements of significantly lower uric acid level in AD [52, 54]. This suggests that metabolic syndrome may interfere with the level of uric acid as an indirect marker of oxidative stress in AD.

#### 3.3.2. Vitamin E

Most of the studies in the literature report that plasma or serum vitamin E level is significantly lower in AD [7, 9, 10, 15, 16, 25, 54, 57–60]. However, vitamin E supplementation does not seem to improve prognosis in AD [61]. So far, erythrocyte and platelet vitamin E levels are not different between AD and controls [15, 29].

#### 3.3.3. Vitamin C

Vitamin C concentration in plasma or serum is also found to be significantly lower in AD in most literature [10, 16, 25, 54, 60, 62, 63]. Furthermore, vitamin C is especially significantly lower in moderate and severe AD [62].

#### 3.3.4. Vitamin A

All of the studies on serum or plasma vitamin A levels establish a significant difference between AD and control [10, 16, 25, 54, 63, 64].

#### 3.3.5. Superoxide Dismutase (SOD)

As an antioxidant enzyme, SOD may be induced or consumed by oxidative stress [65]. It is one of the most studied antioxidant enzymes in AD. Several studies find no difference in SOD between AD and controls [15, 29, 31, 36, 66]. Some find significantly lower erythrocyte level [5, 11, 54, 67] and plasma/serum level [11, 26] in AD, while others find significantly higher erythrocyte [6, 7, 21, 22, 68–71] and plasma [28] levels in AD. However, only three among these studies have a case number more than 80 [7, 22, 68] and all of them either include only mild-to-moderate AD [22, 68] or have clear severity-classification of AD [7]. All three demonstrate significantly higher erythrocyte SOD level in AD [7, 21, 22]. One study further establishes that leukocyte SOD level is higher in moderate AD than in mild AD, higher in mild AD than in controls, and higher in mild AD than in severe AD [7]. This suggests that SOD level is induced by oxidative stress in the early stages of AD and is consumed in the later stage. Studies with different findings may be due to limitations of small sample size [26, 66] or loose inclusion criteria [15, 21, 29, 36, 54].

#### 3.3.6. Glutathione Peroxidase (GPx)/Reduced Glutathione (GSH)

Glutathione peroxidase, another antioxidant enzyme, may be also induced or consumed under conditions of oxidative stress [65]. Some studies demonstrate no difference in GPx level between AD and controls [15, 22, 29, 31]. Some demonstrate lower GPx in AD [26–28, 36, 54], while others observe higher GPx [5, 7, 24, 27]. As for the balance between reduced and oxidized GSH (GSSG), some studies show no significant difference between AD and controls [6, 20, 29], while others demonstrate a balance towards GSSG in AD with statistical significance [7, 32, 41, 72, 73]. The only study with a case number more than 100 demonstrates significantly higher plasma, erythrocyte, and leukocyte GPx and GSSG levels in severe AD than in moderate AD, in moderate AD than in mild AD, and in mild AD than in controls [7]. Other studies may be limited by their small sample size [6, 15, 26] or different patient characteristics [20, 31, 54].

### 3.4. Biomarkers of DNA Oxidation 

8-OHdG is the most commonly studied biomarker for oxidative DNA. Most studies in the literature reveal significantly higher 8-OHdG in AD [4, 16, 74]. Among the three, two studies demonstrated significantly higher peripheral 8-OHdG in lymphocytes and leukocytes [4, 16].

### 3.5. Biomarkers of Central Nervous System

As for CSF antioxidant, the difference of vitamin E level between AD and controls remains controversial [58, 64]. Although one study shows no difference in CSF vitamin C level between AD and controls [75], two other studies reveal that CSF vitamin C level is lower [60, 64] and one study even has negative findings, which may be due to the small sample size [75]. Only one study about CSF vitamin A level shows no difference between AD and control [64].

In terms of markers of oxidative stress measured closer to the brain, one of the studies that measured brain 8-OHdG directly found elevated 8-OHdG in AD [74]. CSF protein carbonyl level is universally significantly higher in AD than in controls [18, 45, 46]. Furthermore, several studies measure protein carbonyls directly in the brain postmortem [14, 43, 47]. Although most studies on the hippocampus have found exclusively significantly higher protein carbonyl levels in AD, one study on other brain regions such as the neocortex, amygdala, brainstem, and cerebellum has also found significantly increased protein carbonyl levels in AD [47].

Regarding nitrated protein that represents protein peroxidation, there is no consistency among different research groups on assessing nitrated protein level in AD. Although direct measurement from brain tissue postmortem reveals consistently significantly higher nitrated protein in AD [19, 43, 49], only two studies demonstrate significantly higher CSF nitrate protein level in AD [46, 48]. Two other studies report no difference between AD and controls [18, 50].

## 4. Conclusions

Most studies show that serum markers of lipid peroxidation are elevated in Alzheimer's disease. However, there is insufficient evidence to justify the routine use of biomarkers as predictors of severity or outcome in AD.

## Figures and Tables

**Table 1:** Studies exploring the predictive capacity of biomarkers of lipid peroxidation in AD.

Reference	Subjects' characteristic	Specimen	Results
TBARS			
Aybek et al. (2007) [20]	62 AD pts (mean age = 73.3, MMSE of 7 to 20 points, ADAS-Cog of 10 to 35 points, GDS >1 and ≤3). 56 controls (mean age = 70.8).	Serum	Significantly higher in AD patients and control group (Mann-Whitney *U* test, *P* = 0.0001).

Casado et al. (2008) [5]	50 AD pts (22 pts aged from 65 to 74, 23 pts aged of 75 to 84, five pts aged >85). Exclusion criteria: DM, myeloproliferative disorders, uncontrolled hypertension, mental retardation, malnutrition. 50 controls (24 subjects age of 65 to 74, 21 subjects age of 75 to 84, five subjects age >85.	Erythrocytes (HPLC)	Significantly higher in AD within group aged 65–74 years (*P* < 0.001), group aged 75–84 years (*P* < 0.001), and group aged 85–94 years (*P* < 0.05).

Bermejo et al. (1997) [13]	18 AD pts (mean age = 76.3). 14 controls (mean age = 75.2).	Erythrocytes (HPLC)	Significantly higher in AD (*P* < 0.01).

Ceballos-Picot et al. (1996) [31]	40 AD pts (mean age = 84.2). Exclusion criteria: life expectancy less than 4 months, taking steroids, blindness, or deafness. 34 controls (mean age = 79.1).	Plasma	No significant difference between AD and controls.

Gironi et al. (2011) [32]	25 AD pts (mean age = 78.2). Exclusion criteria: vascular insult, DM, chronic infection, malignant disease, severe renal, hepatic cardiorespiratory or hematological disease, and use of vitamin supplementation. 66 controls (mean age = 70.4).	Serum	No significant difference between AD and controls.

McGrath et al. (2001) [34]	29 AD pts (mean age = 74, MMSE of 7 to 24). Exclusion criteria: other significant medical problems. 46 controls (mean age 73).	Serum	No significant difference between AD and controls.

Ozcankaya and Delibas (2002) [21]	27 AD pts (mean age = 72.3). Exclusion criteria: life expectancy less than 3 months, taking steroids, blindness and deafness, iron for anemia, illiterate patients, and medical disorder other than dementia. 25 controls (mean age = 64.4).	Serum	Significantly higher in AD (*P* < 0.001).

Serra et al. (2009) [22]	112 AD pts (mean age = 72.1). Exclusion criteria: head trauma, seizures, uncontrolled hypertension, mental retardation, psychosis or depression, smoking within 5 years, and vascular insult. Inclusion: GDS of 3 to 5. 80 controls (mean age = 68.4).	Plasma	Significantly higher in AD (*P* < 0.001).

Torres et al. (2011) [24]	29 AD pts. 26 controls.	Serum	MMSE was negatively associated with MDA levels (*r* = −0.31, *P* = 0.028). Significantly higher in AD (*P* < 0.05).

Cristalli et al. (2012) [7]	110 AD pts (mean age = 74.7, GDS 3 = mild, GDS 4-5 = intermediate, GDS 6-7 = severe). No further exclusion criteria. 134 controls (mean age = 77.8).	Plasma/ erythrocyte/ leukocytes	Significantly higher in mild, intermediate, and severe AD in comparison with control in plasma, erythrocytes and leukocytes samples. Significantly higher in severe AD in comparison with mild and intermediate AD in especially erythrocytes and leukocytes samples. Not inversely correlated with MMSE.

Bourdel-Marchasson et al. (2001) [15]	20 AD pts (mean age = 80.1). Follow-up at memory center for more than 6 months, no evidence of nutritional behavior, mean duration of AD was 3.9 years, 19 pts on cholinergic therapy. 23 controls, mean age 76.0	Plasma/ erythrocytes	Significantly higher in AD in plasma level (*P* = 0.036). No significant difference between AD and control in erythrocytes level.

Polidori et al. (2002) [25]	35 AD pts (mean age = 85.9). 40 controls (mean age = 85.5).	Plasma	Significantly higher in AD in plasma level (*P* < 0.001).

Sekler et al. (2008) [35]	59 AD pts (mean age = 76.4, MMSE = 14.1 ± 6.0). 29 controls (mean age = 70.7).	Plasma	No significant difference between AD and controls.

Serra et al. (2001) [6]	18 AD pts. 14 controls.	Erythrocytes	No significant difference between AD and controls.

Padurariu et al. (2010) [26]	15 AD pts (mean age = 65.8, MMSE = 18.5 ± 0.3, ADAS-cog = 18.5 ± 0.3, not taking antioxidant supplement). 15 controls (mean age = 62.5).	Serum	Significantly higher in AD in serum level (*P* < 0.0005).

Puertas et al. (2012) [28]	20 AD men (mean age = 70.6, MMSE 23.7 ± 0.92). 26 AD women (mean age = 73.9, MMSE 20.7 ± 0.66). Exclusion criteria: taking NSAIDs, steroids, vitamins or antioxidant supplements, history of smoking and alcohol intake, and comorbidity with other clinical major neurological illnesses. 16 control men (mean age = 73.3. 30 control women (mean age = 73.8).	Plasma	Significantly higher in both male and female AD individuals in plasma level (*P* < 0.01).

Ox-LDL			
Aldred et al. (2010) [17]	72 AD pts (mean age = 80, AD Geriatric depression scale = 7.4 ± 3.6, MMSE = 19 ± 4.72). Controls (mean age = 75).	Serum	Significantly higher in AD (*P* = 0.05).

Cai. et al. (2007) [40]	15 AD pts. 15 controls.	Serum	Significantly higher in AD (*P* < 0.05). MMSE inversely correlated with ox-LDL (*P* < 0.05).

**Table 2:** Studies exploring the predictive capacity of biomarkers of protein peroxidation in AD.

Reference	Subjects' characteristic	Specimen	Results
Protein carbonyls			
Cristalli et al. (2012) [7]	110 AD pts (mean age = 74.7, GDS 3 = mild, GDS 4-5 = intermediate, GDS 6-7 = severe). No further exclusion criteria. 134 controls (mean age = 77.8).	plasma/ erythrocyte/ leukocytes	Significantly higher in mild, intermediate, and severe AD in comparison with control in plasma, erythrocytes, and leukocytes samples. Significantly higher in severe AD than intermediate AD and in intermediate AD than mild AD in plasma, erythrocytes, and leukocytes samples. Inversely correlated with MMSE in all kind of samples.

Bermejo et al. (2008) [41]	45 AD pts. 28 controls.	Serum	Significantly higher in AD (*P* < 0.05).

Conrad et al. (2000) [42]	25 AD pts (mean age = 78). 14 controls (mean age = 84).	Serum (HPLC)	Significantly higher in AD (*P* < 0.05).

Aldred et al. (2010) [17]	72 AD pts (mean age = 80, AD GDS = 7.4 ± 3.6, MMSE = 19 ± 4).72 controls (mean age = 75).	Serum	No significant difference between AD and NC.

Cai et al. (2007) [40]	15 AD pts. 15 controls.	Serum	Significantly higher in AD (*P* < 0.05). MMSE inversely correlated with ox-LDL (*P* < 0.05).

Puertas et al. (2012) [28]	20 AD men (mean age = 70.6, MMSE 23.7 ± 0.92). 26 AD women (mean age = 73.9, MMSE 20.7 ± 0.66). Exclusion criteria: taking NSAIDs, steroids, vitamins or antioxidant supplements, history of smoking and alcohol intake, and comorbidity with other clinical major neurological illnesses. 16 control men (mean age = 73.3. 30 control women (mean age = 73.8).	Plasma	Significantly higher in both male and female AD individuals in serum level (*P* < 0.05).

Sultana et al. (2006) [43]	6 AD pts (mean age at death = 84.5, MMSE = 15.7 ± 2.6). 6 controls (mean age at death = 85.8)	Brain: hippocampus	All identified proteins with protein carbonyls significantly higher in AD (*P* ≤ 0.05).

Castegna et al. (2002) [47]	5 AD pts (mean age at death = 85. 5). 5 controls (mean age at death = 83).	Brain: neocortical, hippocampal, entorhinal, amygdala, brainstem, and cerebellum	All identified proteins with protein carbonyls significantly higher in AD (*P* ≤ 0.05).

Korolainen and Pirttilä (2009) [18]	22 AD pts (mean age = 72, 22 AD pts disease duration = 2.3 ± 0.3, MMSE = 21.3 ± 0.9, 12 AD pts APOE4 positive). 10 control APOE4 positive. 18 controls (mean age = 68).	CSF/ plasma/ serum	Significantly higher in all APOE4 negative subjects (*P* = 0.03) and APOE4 negative control (*P* = 0.016) in CSF specimen. Significantly higher in all control subjects than AD in serum (*P* = 0.005). No significant difference in plasma.

Choi et al. (2002) [44]	9 AD pts (age of 70 to 89). 9 controls (age of 74 to 89).	plasma	Significantly higher protein carbonyl in AD (*P* < 0.05).

Korolainen et al. (2007) [45]	11 AD pts (mean age = 73.5, mild AD MMSE of 14 to 26). 8 controls (mean age = 64.6)	CSF	Significantly higher carbonation of *λ* chain precursor in AD (*P* = 0.03).

Ahmed et al. (2005) [46]	32 AD pts (mean age = 71). All before treatment of AchEI. 18 controls (mean age = 69).	CSF	Significantly higher oxidized NFK and DG-H in AD (*P* < 0.05).

Aksenov et al. (2001) [14]	6 AD pts (mean age = 80.4). 6 controls (mean age = 81.1).	Brain: hippocampus, superior and middle temporal lobe	Significantly higher carbonated CKBB and *β*-actin in AD (*P* < 0.05).

Nitrated protein			
Korolainen and Pirttilä (2009) [18]	22 AD pts (mean age = 72, 22 AD pts disease duration = 2.3 ± 0.3, MMSE = 21.3 ± 0.9, 12 AD pts APOE4 positive). 10 control APOE4 positive. 18 controls (mean age = 68).	CSF/ plasma/ serum	No significant difference in CSF, plasma, serum.

Sultana et al. (2006) [43]	6 AD pts (mean age = 84.5). 6 controls (mean age = 85.8).	Brain: hippocampus	All identified proteins with nitrated protein significantly higher in AD (*P* ≤ 0.05).

Tohgi et al. (1999) [48]	25 AD pts (mean age = 67.9, 25 AD pts disease duration = 3.9 ± 2.1, MMSE = 15.6 ± 5.0. 24 controls.	CSF	Significantly higher nitrated protein in AD (*P* = 0.0001). CSF nitrated protein inversely correlated with MMSE.

Castegna et al. (2003) [19]	5 AD pts (mean age at death = 85. 5). 5 controls (mean age at death = 83).	Brain: inferior parietal lobe	A-Enolase, Triosephosphate isomerase, and neuropolypeptide h3 nitration significantly higher in AD (*P* ≤ 0.05).

Hensley et al. (1998) [49]	11 AD pts (mean age at death = 78.5). controls (mean age at death = 78).	Brain: hippocampus, inferior parietal lobe, superior and middle temporal lobe, cerebellum.	Nitrated protein significantly in AD in area of hippocampus, inferior parietal lobe, superior and middle temporal lobe (*P* < 0.05).

Ahmed et al. (2005) [46]	32 AD pts (mean age = 71, all before treatment of AchEI). 18 controls (mean age = 69).	CSF	Significantly higher nitrated protein in AD (*P* < 0.05).

Ryberg et al. (2004) [50]	17 AD pts (mean age = 73.4, mild AD pts MMSE of 19 to 24, moderate AD pts MMSE of 12 to 18, severe AD pts MMSE of 0 to 12. 19 controls (mean age = 68).	CSF	No difference of nitrated protein between AD and control.

**Table 3:** Studies exploring the predictive capacity of antioxidant in AD.

Reference	Subjects' characteristic	Specimen	Results
Antioxidant-uric acid			
Polidori et al. (2002) [25]	35 AD pts (mean age = 85.9). 40 controls (mean age = 85.5).	Plasma	No significant difference between AD and controls.

Carantoni et al. (2000) [8]	24 AD pts (mean age = 83). Exclusion criteria: DM, taking any drug that influences blood glucose and lipid. 66 controls (mean age = 85).	Plasma	No significant difference between AD and controls

Kim et al. (2006) [52]	101 AD pts (mean age = 73.5). Exclusion criteria: DM, hypertension, hyperlipidemia. AD MMSE 17 ± 5.8. 101 controls (mean age = 73.2).	Plasma	Significantly lower in AD (*P* < 0.0001).

Maesaka et al. (1993) [53]	18 AD pts. 11 controls.	Serum	Significantly lower in AD (*P* < 0.02)

Rinaldi et al. (2003) [54]	63 AD pts (mean age = 76.8). Exclusion criteria: smoking, alcohol abuse, major organ failure, dyslipidemia, and malnutrition. 56 controls (mean age = 75.8).	Plasma	Significantly lower in AD (*P* < 0.001)

Antioxidant-vitamin E			
Bourdel-Marchasson et al. (2001) [15]	20 AD pts (mean age = 80.1). Follow-up at memory center for more than 6 months, no evidence of nutritional behavior, mean duration of AD = 3.9 years, 19 pts on cholinergic therapy. 23 controls (mean age = 76.0).	Plasma/ erythrocytes	Significant plasma level in AD (*P* = 0.002). No difference in erythrocytes level.

Cristalli et al. (2012) [7]	110 AD pts (mean age = 74.7, GDS 3 = mild, GDS 4-5 = intermediate, GDS 6-7 = severe). No further exclusion criteria. 134 controls (mean age = 77.8).	Plasma	Significantly lower in mild AD in comparison with control, lower in intermediate AD in comparison with mild AD, and lower in severe AD in comparison with intermediate AD.

Polidori et al. (2002) [25]	35 AD pts (mean age = 85.9). 40 controls (mean age = 85.5).	Plasma	Significantly lower in AD in plasma level (*P* < 0.001).

Jiménez-Jiménez et al. (1997) [58]	44 AD pts. 37 controls.	Serum/ CSF/ serum-CSF ratio	Significantly lower in serum and CSF in AD but no difference in serum-CSF ratio.

Riviere et al. (1998) [62]	44 AD pts (mean age = 78.5, MMSE <24). 20 controls (mean age = 79.3).	Plasma	No significant difference between AD and control.

Rinaldi et al. (2003) [54]	63 AD pts (mean age = 76.8). Exclusion criteria: smoking, alcohol abuse, major organ failure, dyslipidemia, and malnutrition. 56 controls (mean age = 75.8).	Plasma	Significantly lower in AD (*P* < 0.0001)

Iuliano et al. (2010) [57]	37 AD pts (vitamin E levels corrected for cholesterol). 24 controls.	Plasma	Reduced in AD patients (*P* < 0.05).

Schippling et al. (2000) [64]	26 AD pts (mean age = 73.9). 14 controls (mean age = 70.3).	CSF/plasma	No significant difference in CSF and plasma.

Glasø et al. (2004) [60]	20 AD pts. 18 controls.	Serum	Significantly lower in AD (*P* < 0.05).

Sinclair et al. (1998) [9]	25 AD pts (mean age = 74.3, median MMSE = 19). 41 controls (mean age = 73.4).	Plasma	Significantly lower in AD (*P* = 0.035).

Foy et al. (1999) [10]	79 AD pts (median age = 79, MMSE of 10 to 25). 58 controls (median age = 74).	Plasma	Significantly lower in AD (*P* < 0.01).

von Arnim et al. (2012) [63]	74 AD pts. 158 controls.	Serum	No difference between mild dementia and control.

Mecocci et al. (2002) [16]	40 AD pts (mean age = 75.9, mean MMSE = 17.3). 39 controls (mean age = 74.8).	Plasma	Significantly lower in AD (*P* < 0.001).

Antioxidant-vitamin C			
Polidori et al. (2002) [25]	35 AD pts (mean age = 85.9). 40 controls (mean age = 85.5).	plasma	Significantly lower in AD in plasma level (*P* < 0.001).

Riviere et al. (1998) [62]	44 AD pts (mean age = 78.5).	Plasma	Significantly lower in moderate and severe AD (*P* < 0.005)

von Arnim et al. (2012) [63]	74 AD pts. 158 controls	Serum	Significantly lower in demented subjects after adjusting for school education, intake of dietary supplements, smoking habit, body mass index, and alcohol consumption.

Rinaldi et al. (2003) [54]	63 AD pts (mean age = 76.8). Exclusion criteria: smoking, alcohol abuse, major organ failure, dyslipidemia, and malnutrition. 56 controls (mean age = 75.8).	Plasma	Significantly lower in AD (*P* < 0.0001)

Glaso et al. (2004) [60]	20 AD pts. 18 controls.	Serum/CSF	Significantly lower in AD (*P* < 0.05).

Sinclair et al. (1998) [9]	25 AD pts (mean age = 74.3, AD median MMSE = 19). 41 controls (mean age = 73.4).	Plasma	No significant difference between AD and control.

Foy et al. (1999) [10]	79 AD pts (median age = 79, MMSE of 10 to 25). 58 controls (median age = 74).	Plasma	Significantly lower in AD (*P* < 0.001).

Paraskevas et al. (1997) [75]	17 AD pts. 15 controls.	Plasma/CSF	No significant difference.

Schippling et al. (2000) [64]	26 AD pts (mean age = 73.9). 14 controls (mean age = 70.3).	CSF/plasma	No significant difference in plasma, but significantly lower in CSF (*P* < 0.01).

Mecocci et al. (2002) [16]	40 AD pts (mean age = 75.9, mean MMSE = 17.3). 39 controls (mean age = 74.8).	Plasma/	Significantly lower in AD (*P* < 0.001).

Antioxidant-vitamin A/carotene			
Polidori et al. (2002) [25]	35 AD pts (mean age = 85.9). 40 controls (mean age = 85.5).	plasma	Significantly lower in AD in plasma level (*P* < 0.001).

von Arnim et al. (2012) [63]	74 AD pts. 158 controls.	Serum	Significantly lower in demented subjects after adjusting for school education, intake of dietary supplements, smoking habit, body mass index, and alcohol consumption.

Rinaldi et al. (2003) [54]	63 AD pts (mean age = 76.8). Exclusion criteria: smoking, alcohol abuse, major organ failure, dyslipidemia, and malnutrition. 56 controls (mean age = 75.8).	Plasma	Significantly lower in AD (*P* < 0.001)

Schippling et al. (2000) [64]	26 AD pts (mean age = 73.9). 14 controls (mean age = 70.3).	CSF/plasma	No significant difference in CSF but significantly lower in plasma (*P* < 0.001).

Sinclair et al. (1998) [9]	25 AD pts (mean age = 74.3, median MMSE = 19). 41 controls (mean age = 73.4).	Plasma	No significant difference between AD and control.

Foy et al. (1999) [10]	79 AD pts (median age = 79, MMSE of 10 to 25). 58 controls (median age = 74).	Plasma	Significantly lower in AD (*P* < 0.01).

Mecocci et al. (2002) [16]	40 AD pts (mean age = 75.9, mean MMSE = 17.3). 39 controls (mean age = 74.8)	Plasma/	Significantly lower in AD (*P* < 0.001).

Antioxidant-SOD			
Casado et al. (2008) [5]	50 AD pts (22 pts aged from 65 to 74, 23 pts aged from 75 to 84, five pts aged >85). Exclusion criteria: DM, myeloproliferative disorders, uncontrolled hypertension, mental retardation, and malnutrition. 50 controls (24 subjects aged from 65 to 74, 21 subjects aged from 75 to 84, five subjects aged >85).	Erythrocytes (HPLC)	Significantly lower in AD within group aged 65–74 years (*P* < 0.02), group aged 75–84 years (*P* < 0.005), and group aged 85–94 years (*P* < 0.001).

Jeandel et al. (1989) [36]	55 AD pts. 24 controls.	Erythrocytes	No significant difference between AD and controls.

Ceballos-Picot et al. (1996) [31]	40 AD pts (mean age = 84.2). Exclusion criteria: life expectancy less than 4 months, taking steroids, and blindness or deafness. 34 controls (mean age = 79.1).	Erythrocytes	No significant difference between AD and controls.

Ozcankaya and Delibas (2002) [21]	27 AD pts (mean age = 72.3). Exclusion criteria: life expectancy less than 3 months, taking steroids, blindness and deafness, iron for anemia, illiterate patients, and medical disorder other than dementia. 25 controls (mean age = 64.4).	Erythrocytes	Significantly higher in AD (*P* < 0.001).

Serra et al. (2009) [22]	112 AD pts (mean age = 72.1). Exclusion criteria: head trauma, seizures, uncontrolled hypertension, mental retardation, psychosis or depression, smoking within 5 years, and vascular insult. Inclusion criteria: GDS of 3 to 5. 80 controls (mean age = 68.4).	Erythrocytes	Significantly higher in AD (*P* < 0.001).

Cristalli et al. (2012) [7]	110 AD pts (mean age = 74.7, GDS 3 = mild, GDS 4-5 = intermediate, GDS 6-7 = severe). No further exclusion criteria. 134 controls (mean age = 77.8).	Erythrocyte/ leukocytes	Significantly higher in mild, intermediate, and severe AD in comparison with control in erythrocytes and leukocytes samples. Significantly higher in severe AD than intermediate AD, higher in intermediate AD than mild AD in erythrocyte sample (*P* < 0.05). Significantly higher in intermediate AD than mild AD, higher in mild AD than severe AD in leukocyte sample (*P* < 0.05).

Bourdel-Marchasson et al. (2001) [15]	20 AD pts (mean age = 80.1). Follow-up at memory center for more than 6 months, no evidence of nutritional behavior, mean duration of AD = 3.9 years, 19 pts on cholinergic therapy. 23 controls (mean age = 76.0).	Erythrocytes	No significant difference between AD and controls.

Serra et al. (2001) [6]	18 AD pts. 14 controls.	Erythrocytes	Significantly higher in AD (*P* = 0.0000001).

Padurariu et al. (2010) [26]	15 AD pts (mean age = 65.8, MMSE = 18.5 ± 0.3, ADAS-cog = 18.5 ± 0.3, not taking antioxidant supplement). 15 controls (mean age = 62.5)	Serum	Significantly lower in AD in serum level (*P* < 0.0004).

Puertas et al. (2012) [28]	20 AD men (mean age = 70.6, MMSE 23.7 ± 0.92). 26 AD women (mean age = 73.9, MMSE 20.7 ± 0.66). Exclusion criteria: taking NSAIDs, steroids, vitamins or antioxidant supplements, history of smoking and alcohol intake, and comorbidity with other clinical major neurological illnesses. 16 control men (mean age = 73.3). 30 control women (mean age = 73.8).	Plasma	Significantly higher in total AD in plasma level (*P* < 0.01), but not in men and women separately.

Rinaldi et al. (2003) [54]	63 AD pts (mean age = 76.8). Exclusion criteria: smoking, alcohol abuse, major organ failure, dyslipidemia, and malnutrition. 56 controls (mean age = 75.8).	Erythrocytes	Significantly lower in AD (*P* < 0.0001).

Ihara et al. (1997) [11]	22 AD pts (mean age = 74.8). 19 controls (mean age = 73.5).	Erythrocytes/ plasma	Significantly lower in AD (*P* < 0.001).

Kharrazi et al. (2008) [68]	91 AD pts (mean age = 75, MMSE = 18.4 ± 4.8). 91 controls (mean age = 73.5)	Erythrocytes	Significantly higher in AD (*P* = 0.001).

Perrin et al. (1990) [69]	25 AD pts. 25 controls.	Erythrocytes	Significantly higher in AD (*P* < 0.05).

Rossi et al. (2002) [70]	32 AD pts (mean age = 72, MMSE = 19.2 ± 5.8). 22 controls (mean age = 70).	Erythrocytes	Significantly higher in AD (*P* = 0.014).

Snaedal et al. (1998) [67]	44 AD pts. 44 controls.	Erythrocytes	Significantly lower in AD (*P* = 0.019).

Tabet et al. (2001) [66]	7 AD pts (mean age = 75, MMSE 21 ± 1.6). Exclusion criteria: other physical illnesses/treatments that could influence free radical or antioxidant levels (e.g., cancer, radiation therapy, oxygen intoxication, or liver disease). 6 controls (mean age = 71).	Erythrocytes	No significant difference.

De Leo et al. (1998) [71]	31 AD pts (mean age at 65.4, MMSE >16). 21 controls (mean age = 66.2).	Erythrocytes	Significantly higher in AD (*P* < 0.01).

Glutathione peroxidase/glutathione reductase/reduced glutathione			
Aybek et al. (2007) [20]	62 AD pts (mean age = 73.3, MMSE of 7 to 20, ADAS-cog of 10 to 35, GDS of 1 to 3. 56 controls (mean age = 70.8).	Serum	No significant difference of reduced GSH between AD and control.

Casado et al. (2008) [5]	50 AD pts (22 pts aged from 65 to 74, 23 pts aged from 75 to 84, five pts aged >85). Exclusion criteria: DM, myeloproliferative disorders, uncontrolled hypertension, mental retardation, and malnutrition. 50 controls (24 subjects aged from 65 to 74, 21 subjects aged from 75 to 84, five subjects aged >85.	Erythrocytes (HPLC)	Significantly higher glutathione peroxidase (GPX) in AD within group aged 65–74 years (*P* < 0.005) and group aged 75–84 years (*P* < 0.005), but not within group aged 85–94 years.

Ceballos-Picot et al. (1996) [31]	40 AD pts (mean age = 84.2). Exclusion criteria: life expectancy less than 4 months, taking steroids, and blindness or deafness. 34 controls (mean age = 79.1).	Plasma	No significant difference of GPX between AD and controls.

Jeandel et al. (1989) [36]	55 AD pts. 24 controls.	Erythrocytes	Significantly lower of GPX in AD (*P* < 0.05).

Gironi et al. (2011) [32]	25 AD pts (mean age = 78.2). Inclusion age of 65 to 90. Exclusion criteria: vascular insult, DM, chronic infection, malignant disease, severe renal, hepatic cardiorespiratory or hematological disease, and use of vitamin supplementation. 66 controls (mean age = 70.4).	Serum	Significantly lower in reduced GSH in AD (*P* < 0.05).

Serra et al. (2009) [22]	112 AD pts (mean age = 72.1). Exclusion criteria: head trauma, seizures, uncontrolled hypertension, mental retardation, psychosis or depression, smoking within 5 years, and vascular insult. Inclusion criteria: GDS of 3 to 5. 80 controls (mean age = 68.4).	Plasma	No significant difference of GPX.

Torres et al. (2011) [24]	29 AD pts. 26 controls	Serum	Significantly higher GPX in AD (*P* < 0.05).

Cristalli et al. (2012) [7]	110 AD pts (mean age = 74.7, GDS 3 = mild, GDS 4-5 = intermediate, GDS 6-7 = severe). No further exclusion criteria. 134 controls (mean age = 77.8).	Plasma/ erythrocyte/ leukocytes	Significantly higher oxidized GSH (GSSG) in severe AD than intermediate AD, intermediate AD than mild AD, and mild AD than control. Significantly lower GSH/GSSG in similar pattern. All of these findings present plasma, erythrocytes. Significantly higher GPX in severe AD than intermediate AD, intermediate AD than mild AD, and mild AD than control.

Bourdel-Marchasson et al. (2001) [15]	20 AD pts (mean age = 80.1). Follow-up at memory center for more than 6 months, no evidence of nutritional behavior, mean duration of AD = 3.9 years, 19 pts on cholinergic therapy. 23 controls (mean age = 76.0)	Plasma/ erythrocytes	No significant difference of GPX between AD and control in erythrocytes level.

Serra et al. (2001) [6]	18 AD pts. 14 controls.	Erythrocytes	No significant difference of GSH between AD and controls.

Padurariu et al. (2010) [26]	15 AD pts (mean age = 65.8, MMSE = 18.5 ± 0.3, ADAS-cog = 18.5 ± 0.3, not taking antioxidant supplement). 15 controls (mean age = 62.5).	Serum	Significantly lower GPX in AD in serum level (*P* < 0.0001).

Puertas et al. (2012) [28]	20 AD men (mean age = 70.6, MMSE = 23.7 ± 0.92). 26 AD women (mean age = 73.9, MMSE = 20.7 ± 0.66). Exclusion criteria: taking NSAIDs, steroids, vitamins or antioxidant supplements, history of smoking and alcohol intake, and comorbidity with other clinical major neurological illnesses. 16 control men (mean age = 73.3). 30 control women (mean age = 73.8).	Plasma	Significantly lower GPX in both male and female AD in plasma level (*P* < 0.01).

Bermejo et al. (2008) [41]	45 AD pts. 28 controls.	Serum	Significantly lower GSH/GSSG in AD (*P* < 0.05).

Rinaldi et al. (2003) [54]	63 AD pts (mean age = 76.8). Exclusion criteria: smoking, alcohol abuse, major organ failure, dyslipidemia, and malnutrition. 56 controls (mean age = 75.8).	Plasma	Significantly lower GPX in AD (*P* < 0.0001).

Baldeiras et al. (2008) [73]	42 AD pts. 37 controls.	Plasma	Significantly higher GSSG in AD (*P* < 0.05).

Vural et al. (2010) [76]	50 AD pts (mean age = 71.9). Exclusion criteria: drug abuse, DM, hypertension, severe head injury, and seizure disorder. 50 controls (mean age = 65.1).	Plasma	Significantly lower GPX in AD (*P* < 0.01).

8-hydroxyguanosine			
Mecocci et al. (2002) [16]	40 AD pts (mean age = 75.9, MMSE = 17.3 ± 2.1). 39 controls (mean age = 74.8).	Lymphocytes	Significantly higher in AD (*P* < 0.001).

Migliore et al. (2005) [4]	20 AD pts (mean age = 71.05, AD disease duration = 3.3 ± 1.53). 15 controls (mean age = 65.8).	Leukocytes	Significantly higher in AD (*P* < 0.001).

Nunomura et al. (2012) [74]	15 AD pts (mean age = 89.5). Pathologically definite AD classified into preclinical, MCI, and mild AD. 5 controls (mean age = 88.4).	Brain	Significantly higher in mild AD and MCI than preclinical and control.

GDS: geriatric depression scale; CSF: cerebrospinal fluid; CDR: clinical dementia rating; DM: diabetes mellitus; MMSE: mini-mental state examination; AD: Alzheimer's disease; AchEI: anticholinesterase inhibitor; and pts: patients.

## References

[1] Weiner MW, Veitch DP, Aisen PS (2012). The Alzheimer’s disease neuroimaging initiative: a review of papers published since its inception. *Alzheimer’s and Dementia*.

[2] Hardy JA, Higgins GA (1992). Alzheimer’s disease: the amyloid cascade hypothesis. *Science*.

[3] Smith MA, Rottkamp CA, Nunomura A, Raina AK, Perry G (2000). Oxidative stress in Alzheimer's disease. *Biochimica et Biophysica Acta*.

[4] Migliore L, Fontana I, Trippi F (2005). Oxidative DNA damage in peripheral leukocytes of mild cognitive impairment and AD patients. *Neurobiology of Aging*.

[5] Casado Á, Encarnación López-Fernández M, Concepción Casado M, De La Torre R (2008). Lipid peroxidation and antioxidant enzyme activities in vascular and alzheimer dementias. *Neurochemical Research*.

[6] Serra JA, Domínguez RO, De Lustig ES (2001). Parkinson’s disease is associated with oxidative stress: comparison of peripheral antioxidant profiles in living Parkinson’s, Alzheimer’s and vascular dementia patients. *Journal of Neural Transmission*.

[7] Cristalli DO, Arnal N, Marra FA, De Alaniz MJT, Marra CA (2012). Peripheral markers in neurodegenerative patients and their first-degree relatives. *Journal of the Neurological Sciences*.

[8] Carantoni M, Zuliani G, Munari MR, D’Elia K, Palmieri E, Fellin R (2000). Alzheimer disease and vascular dementia: relationships with fasting glucose and insulin levels. *Dementia and Geriatric Cognitive Disorders*.

[9] Sinclair AJ, Bayer AJ, Johnston J, Warner C, Maxwell SR (1998). Altered plasma antioxidant status in subjects with Alzheimer's disease and vascular dementia. *International Journal of Geriatric Psychiatry*.

[10] Foy CJ, Passmore AP, Vahidassr MD, Young IS, Lawson JT (1999). Plasma chain-breaking antioxidants in Alzheimer’s disease, vascular dementia and Parkinson’s disease. *QJM*.

[11] Ihara Y, Hayabara T, Sasaki K (1997). Free radicals and superoxide dismutase in blood of patients with Alzheimer’s disease and vascular dementia. *Journal of the Neurological Sciences*.

[12] Guidi I, Galimberti D, Lonati S (2006). Oxidative imbalance in patients with mild cognitive impairment and Alzheimer’s disease. *Neurobiology of Aging*.

[13] Bermejo P, Gomez-Serranillos P, Santos J, Pastor E, Gil P, Martin-Aragon S (1997). Determination of malonaldehyde in Alzheimer’s disease: a comparative study of high-performance liquid chromatography and thiobarbituric acid test. *Gerontology*.

[14] Aksenov MY, Aksenova MV, Butterfield DA, Geddes JW, Markesbery WR (2001). Protein oxidation in the brain in Alzheimer’s disease. *Neuroscience*.

[15] Bourdel-Marchasson I, Delmas-Beauviex M-C, Peuchant E (2001). Antioxidant defences and oxidative stress markers in erythrocytes and plasma from normally nourished elderly Alzheimer patients. *Age and Ageing*.

[16] Mecocci P, Cristina Polidori M, Cherubini A (2002). Lymphocyte oxidative DNA damage and plasma antioxidants in Alzheimer disease. *Archives of Neurology*.

[17] Aldred S, Bennett S, Mecocci P (2010). Increased low-density lipoprotein oxidation, but not total plasma protein oxidation, in Alzheimer’s disease. *Clinical Biochemistry*.

[18] Korolainen MA, Pirttilä T (2009). Cerebrospinal fluid, serum and plasma protein oxidation in Alzheimer’s disease. *Acta Neurologica Scandinavica*.

[19] Castegna A, Thongboonkerd V, Klein JB, Lynn B, Markesberyl WR, Butterfield DA (2003). Proteomic identification of nitrated proteins in Alzheimer’s disease brain. *Journal of Neurochemistry*.

[20] Aybek H, Ercan F, Aslan D, Şahiner T (2007). Determination of malondialdehyde, reduced glutathione levels and APOE4 allele frequency in late-onset Alzheimer’s disease in Denizli, Turkey. *Clinical Biochemistry*.

[21] Ozcankaya R, Delibas N (2002). Malondialdehyde, superoxide dismutase, melatonin, iron, copper, and zinc blood concentrations in patients with Alzheimer disease: cross-sectional study. *Croatian Medical Journal*.

[22] Serra JA, Domínguez RO, Marschoff ER, Guareschi EM, Famulari AL, Boveris A (2009). Systemic oxidative stress associated with the neurological diseases of aging. *Neurochemical Research*.

[23] Sinem F, Dildar K, Gökhan E, Melda B, Orhan Y, Filiz M (2010). The serum protein and lipid oxidation marker levels in Alzheimer’s disease and effects of cholinesterase inhibitors and antipsychotic drugs therapy. *Current Alzheimer Research*.

[24] Torres LL, Quaglio NB, De Souza GT (2011). Peripheral oxidative stress biomarkers in mild cognitive impairment and Alzheimer’s disease. *Journal of Alzheimer’s Disease*.

[25] Polidori MC, Mecocci P (2002). Plasma susceptibility to free radical-induced antioxidant consumption and lipid peroxidation is increased in very old subjects with Alzheimer disease. *Journal of Alzheimer’s Disease*.

[26] Padurariu M, Ciobica A, Hritcu L, Stoica B, Bild W, Stefanescu C (2010). Changes of some oxidative stress markers in the serum of patients with mild cognitive impairment and Alzheimer’s disease. *Neuroscience Letters*.

[27] Martín-Aragón S, Bermejo-Bescós P, Benedí J (2009). Metalloproteinase’s activity and oxidative stress in mild cognitive impairment and Alzheimer’s disease. *Neurochemical Research*.

[28] Puertas MC, Martínez-Martos JM, Cobo MP, Carrera MP, Mayas MD, Rami'rez-Expósito MJ (2012). Plasma oxidative stress parameters in men and women with early stage Alzheimer type dementia. *Experimental Gerontology*.

[29] Fernandes MA, Proenca MT, Nogueira AJ (1999). Influence of apolipoprotein E genotype on blood redox status of Alzheimer’s disease patients. *International Journal of Molecular Medicine*.

[30] Galbusera C, Facheris M, Magni F (2004). Increased susceptibility to plasma lipid peroxidation in Alzheimer disease patients. *Current Alzheimer Research*.

[31] Ceballos-Picot I, Merad-Boudia M, Nicole A (1996). Peripheral antioxidant enzyme activities and selenium in elderly subjects and in dementia of Alzheimer’s type—place of the extracellular glutathione peroxidase. *Free Radical Biology and Medicine*.

[32] Gironi M, Bianchi A, Russo A (2011). Oxidative imbalance in different neurodegenerative diseases with memory impairment. *Neurodegenerative Diseases*.

[33] Kalman J, Dey I, Ilona SV (1994). Platelet membrane fluidity and plasma malondialdehyde levels in Alzheimer’s demented patients with and without family history of dementia. *Biological Psychiatry*.

[34] McGrath LT, McGleenon BM, Brennan S, McColl D, McIlroy S, Passmore AP (2001). Increased oxidative stress in Alzheimer’s disease as assessed with 4-hydroxynonenal but not malondialdehyde. *QJM*.

[35] Sekler MA, Jiménez JM, Rojo L (2008). Cognitive impairment and Alzheimer’s disease: links with oxidative stress and cholesterol metabolism. *Neuropsychiatric Disease and Treatment*.

[36] Jeandel C, Nicolas MB, Dubois F, Nabet-Belleville F, Penin F, Cuny G (1989). Lipid peroxidation and free radical scavengers in Alzheimer’s disease. *Gerontology*.

[37] Schrag M, Mueller C, Zabel M (2013). Oxidative stress in blood in Alzheimer's disease and mild cognitive impairment: a meta-analysis. *Neurobiology of Disease*.

[38] Uno M, Kitazato KT, Nishi K, Itabe H, Nagahiro S (2003). Raised plasma oxidised LDL in acute cerebral infarction. *Journal of Neurology Neurosurgery and Psychiatry*.

[39] Teunissen CE, De Vente J, Steinbusch HWM, De Bruijn C (2002). Biochemical markers related to Alzheimer’s dementia in serum and cerebrospinal fluid. *Neurobiology of Aging*.

[40] Cai Z-Y, Yan Y, Yan L (2007). Serum level of MMP-2, MMP-9 and Ox-LDL in Alzheimer’s disease with hyperlipoidemia. *Journal of Medical Colleges of PLA*.

[41] Bermejo P, Martín-Aragón S, Benedí J (2008). Peripheral levels of glutathione and protein oxidation as markers in the development of Alzheimer’s disease from Mild Cognitive Impairment. *Free Radical Research*.

[42] Conrad CC, Marshall PL, Talent JM, Malakowsky CA, Choi J, Gracy RW (2000). Oxidized proteins in Alzheimer's plasma. *Biochemical and Biophysical Research Communications*.

[43] Sultana R, Poon HF, Cai J (2006). Identification of nitrated proteins in Alzheimer’s disease brain using a redox proteomics approach. *Neurobiology of Disease*.

[44] Choi J, Malakowsky CA, Talent JM, Conrad CC, Gracy RW (2002). Identification of oxidized plasma proteins in Alzheimer's disease. *Biochemical and Biophysical Research Communications*.

[45] Korolainen MA, Nyman TA, Nyyssönen P, Hartikainen ES, Pirttilä T (2007). Multiplexed proteomic analysis of oxidation and concentrations of cerebrospinal fluid proteins in Alzheimer disease. *Clinical Chemistry*.

[46] Ahmed N, Ahmed U, Thornalley PJ, Hager K, Fleischer G, Münch G (2005). Protein glycation, oxidation and nitration adduct residues and free adducts of cerebrospinal fluid in Alzheimer’s disease and link to cognitive impairment. *Journal of Neurochemistry*.

[47] Castegna A, Aksenov M, Aksenova M (2002). Proteomic identification of oxidatively modified proteins in Alzheimer’s disease brain. Part I: creatine kinase BB, glutamine synthase, and ubiquitin carboxy-terminal hydrolase L-1. *Free Radical Biology and Medicine*.

[48] Tohgi H, Abe T, Yamazaki K, Murata T, Ishizaki E, Isobe C (1999). Alterations of 3-nitrotyrosine concentration in the cerebrospinal fluid during aging and in patients with Alzheimer’s disease. *Neuroscience Letters*.

[49] Hensley K, Maidt ML, Yu Z, Sang H, Markesbery WR, Floyd RA (1998). Electrochemical analysis of protein nitrotyrosine and dityrosine in the Alzheimer brain indicates region-specific accumulation. *Journal of Neuroscience*.

[50] Ryberg H, Söderling A-S, Davidsson P, Blennow K, Caidahl K, Persson LI (2004). Cerebrospinal fluid levels of free 3-nitrotyrosine are not elevated in the majority of patients with amyotrophic lateral sclerosis or Alzheimer’s disease. *Neurochemistry International*.

[51] Polidori MC, Stahl W, Eichler O, Niestroj I, Sies H (2001). Profiles of antioxidants in human plasma. *Free Radical Biology and Medicine*.

[52] Kim T-S, Pae C-U, Yoon S-J (2006). Decreased plasma antioxidants in patients with Alzheimer’s disease. *International Journal of Geriatric Psychiatry*.

[53] Maesaka JK, Wolf-Klein G, Piccione JM, Ma CM (1993). Hypouricemia, abnormal renal tubular urate transport, and plasma natriuretic factor(s) in patients with Alzheimer’s disease. *Journal of the American Geriatrics Society*.

[54] Rinaldi P, Polidori MC, Metastasio A (2003). Plasma antioxidants are similarly depleted in mild cognitive impairment and in Alzheimer’s disease. *Neurobiology of Aging*.

[55] Ruggiero C, Cherubini A, Lauretani F (2009). Uric acid and dementia in community-dwelling older persons. *Dementia and Geriatric Cognitive Disorders*.

[56] Schretlen DJ, Inscore AB, Jinnah HA, Rao V, Gordon B, Pearlson GD (2007). Serum uric acid and cognitive function in community-dwelling older adults. *Neuropsychology*.

[57] Iuliano L, Monticolo R, Straface G (2010). Vitamin E and enzymatic/oxidative stress-driven oxysterols in amnestic mild cognitive impairment subtypes and Alzheimer’s disease. *Journal of Alzheimer’s Disease*.

[58] Jiménez-Jiménez FJ, de Bustos F, Molina JA (1997). Cerebrospinal fluid levels of alpha-tocopherol (vitamin E) in Alzheimer's disease. *Journal of Neural Transmission*.

[59] Zaman Z, Roche S, Fielden P, Frost PG, Niriella DC, Cayley ACD (1992). Plasma concentrations of vitamins A and E and carotenoids in Alzheimer’s disease. *Age and Ageing*.

[60] Glasø M, Nordbø G, Diep L, Bøhmer T (2004). Reduced concentrations of several vitamins in normal weight patients with late-onset dementia of the Alzheimer type without vascular disease. *Journal of Nutrition, Health and Aging*.

[61] Sano M, Ernesto C, Thomas RG (1997). A controlled trial of selegiline, alpha-tocopherol, or both as treatment for Alzheimer’s disease. *The New England Journal of Medicine*.

[62] Riviere S, Birlouez-Aragon I, Nourhashémi F, Vellas B (1998). Low plasma vitamin C in Alzheimer patients despite an adequate diet. *International Journal of Geriatric Psychiatry*.

[63] von Arnim CA, Herbolsheimer F, Nikolaus T (2012). Dietary antioxidants and dementia in a population-based case-control study among older people in South Germany. *Journal of Alzheimer's Disease*.

[64] Schippling S, Kontush A, Arlt S (2000). Increased lipoprotein oxidation in Alzheimer’s disease. *Free Radical Biology and Medicine*.

[65] Cherubini A, Ruggiero C, Polidori MC, Mecocci P (2005). Potential markers of oxidative stress in stroke. *Free Radical Biology and Medicine*.

[66] Tabet N, Mantle D, Walker Z, Orrell M (2001). Vitamins, trace elements, and antioxidant status in dementia disorders. *International Psychogeriatrics*.

[67] Snaedal J, Kristinsson J, Gunnarsdóttir S, Ólafsdóttir Á, Baldvinsson M, Jóhannesson T (1998). Copper, ceruloplasmin and superoxide dismutase in patients with Alzheimer’s disease. A case-control study. *Dementia and Geriatric Cognitive Disorders*.

[68] Kharrazi H, Vaisi-Raygani A, Rahimi Z, Tavilani H, Aminian M, Pourmotabbed T (2008). Association between enzymatic and non-enzymatic antioxidant defense mechanism with apolipoprotein E genotypes in Alzheimer disease. *Clinical Biochemistry*.

[69] Perrin R, Briancon S, Jeandel C (1990). Blood activity of Cu/Zn superoxide dismutase, glutathione peroxidase and catalase in Alzheimer’s disease: a case-control study. *Gerontology*.

[70] Rossi L, Squitti R, Pasqualetti P (2002). Red blood cell copper, zinc superoxide dismutase activity is higher in Alzheimer’s disease and is decreased by D-penicillamine. *Neuroscience Letters*.

[71] De Leo ME, Borrello S, Passantino M (1998). Oxidative stress and overexpression of manganese superoxide dismutase in patients with Alzheimer’s disease. *Neuroscience Letters*.

[72] Bowes MP, Zivin JA, Thomas GR, Thibodeaux H, Fagan SC (1996). Acute hypertension, but not thrombolysis, increases the incidence and severity of hemorrhagic transformation following experimental stroke in rabbits. *Experimental Neurology*.

[73] Baldeiras I, Santana I, Proença MT (2008). Peripheral oxidative damage in mild cognitive impairment and mild Alzheimer’s disease. *Journal of Alzheimer’s Disease*.

[74] Nunomura A, Tamaoki T, Motohashi N (2012). The earliest stage of cognitive impairment in transition from normal aging to alzheimer disease is marked by prominent RNA oxidation in vulnerable neurons. *Journal of Neuropathology and Experimental Neurology*.

[75] Paraskevas GP, Kapaki E, Libitaki G, Zournas C, Segditsa I, Papageorgiou C (1997). Ascorbate in healthy subjects, amyotrophic lateral sclerosis and Alzheimer’s disease. *Acta Neurologica Scandinavica*.

[76] Vural H, Demirin H, Kara Y, Eren I, Delibas N (2010). Alterations of plasma magnesium, copper, zinc, iron and selenium concentrations and some related erythrocyte antioxidant enzyme activities in patients with Alzheimer’s disease. *Journal of Trace Elements in Medicine & Biology*.

